# Pilocarpin in the Treatment of Malarial Fever

**Published:** 1880-12-20

**Authors:** Gaspar Griswold


### PILOCARPIN IN THE TREATMENT OF MALARIAL FEVER.

* * The salts of quinia are considered specific in malarial affections, and their efficacy is beyond question, but in many cases they do not act promptly enough. What is needed is an agency that will antagonize the essential conditions of a chill at once; which, given during a chill, will cut it short, and which, given just as a chill is threatening, will prevent its occurrence. That the profession has felt the want of such an agent is well-attested by the long array of measures which have been proposed for the purpose. Sinapisms all over the body, the application of tourniquets to the limbs, cups (wet and dry) to the spine, the full administration of alcohol, narcotic doses of opium, and drachm-doses of chloroform internally, are some of them. These methods either have proved so uncertain, or involve so much discomfort, that at the present day treatment of the paroxysm is nearly always merely palliative, being simply an attempt to make the patient as comfortable as possible while he is passing through the different stages.

The essential conditions of a chill are a small, hard pulse, peripheral anæmia, and convulsive muscular contractions. Pilocarpin relaxes arterial tension, causes a determination of blood to the surface, and in the progress of the diaphoresis induced by it brings about muscular relaxation. It is easy to see how pilocarpin, sending the blood to the surface and causing diaphoresis within two or three minutes from the time of its hypodermic administration, can quickly dispel the blue, shrunken appearance of the skin and cold, creeping sensations which are about to usher in a chill; just as nitrate of amyl, dilating the cerebral vessels, prevents the epileptic seizure which their spasmodic contraction is on the point of causing.

To subject this reasoning to a clinical test, six cases of malarial intermittent fever were selected. Each patient was carefully watched at the time when his paroxysm was due, and two or three minutes after the chill had fairly begun gr. 1-5th of the muriate of pilocarpin was administered hypodermically. The patient's temperature was then taken every thirty minutes for the next four or five hours. The results were as follows: 1. In all but one case the chill stopped within two or three minutes after the pilocarpin was administered; and the paroxysms aborted, terminating in the sweat caused by the medicine—no hot stage occurring. In the remaining case, the patient was a very large man, and the dose administered did not produce marked diaphoresis; the chill was not interrupted, although its severity was diminished, and the pains in the back and loins disappeared. A hot stage occurred, but was shorter and less intense than that of the preceding paroxysm. Perhaps a more profuse diaphoresis might have been successful in this case, as it was in the others. With this idea it was proposed, in case another paroxysm occurred, to give a larger dose of pilocarpin; unfortunately for the settlement of this question, if not for himself, the patient recovered without having another chill. In one of these cases quinine also was given; in the others pilocarpin was the only remedy. 2. In all the cases recovery followed the administration of a single dose of pilocarpin; in no instance did another chill occur. In a seventh case small doses of quinine (five grains three times a day) were prescribed; a chill, threatening to develop, was anticipated and prevented with pilocarpin. Convalescence was established without the occurrence of another paroxysm.

From these cases it seems fair to conclude: 1. That the muriate of pilocarpin, administered hypodermically, will promptly cut short the chill of malarial intermittent fever. 2. That in a large proportion of cases so treated the paroxysm aborts, terminating in the sweat caused by the pilocarpin, there being no hot stage. 3. That such abortion of a paroxysm is in itself sufficient to effect a cure in many cases. 4. That such abortion of a paroxysm is a valuabie adjuvant to treatment with quinine during the intervals. 5. That a dose of pilocarpin sufficient to produce this effect acts gently, without causing exhausting diaphoresis or unpleasant ptyalism. 6. That the promptness with which an adequate dose of pilocarpin interrupts a chill is suggestive of its possible efficacy in cases of pernicious intermittent fever, where prevention of the full development of a paroxysm is often of the first importance.

It will be observed that in all but one of the preceding cases the medicine was administered *during the chill.* I have more lately been able to study seventeen cases in which pilocarpin was given *before the chill*, with a view to preventing its occurrence. In five of these the remedy was administered hypodermically; diaphoresis resulted in from two to five minutes, and in every case the chill was prevented. In one of these cases a second dose was required two days afterward, which was again successful. Quinine was given, in three instances, in small doses (three to five grains three times a day). In the twelve remaining cases the muriate of pilocarpin was given by the mouth. In two instances the medicine failed to act, no diaphoresis being produced; in these cases the impending paroxysms were not prevented, but went through their usual course. In ten instances diaphoresis, more or less marked, resulted in from ten to twenty minutes after the pilocarpin was taken; in all these cases the paroxysm was averted. In three of these twelve cases, including the two in which no diaphoresis was produced, it was found necessary to use pilocarpin again. About half of the twelve patients took quinine.

Administered hypodermically, the drug acts more surely, more rapidly, more evenly; the dose required varies between gr. 1-5th and gr. 1-6th, according as the patient is large or below medium size. The following solution may be used:

℞ Pilocarpiæ muriat....................................gr. j.

Aquæ destill.............................................ʒ j.

Sig. m. x=gr. 1-6th. M.

Like similar solutions of other alkaloids, this one begins to lose strength' and is no longer reliable, after standing two or three weeks in a warm room. One-grain powders of the drug may be kept for an indefinite time, put up by the druggist in a manner to prevent deliquescence; the above-mentioned solution can then be made fresh as occasion may require.

If the patient objects to hypodermic medication, or if circumstances render this method of administration inconvenient, the remedy may be given by the mouth, and yet act efficiently. In this case the dose will vary between gr. 1-4th and gr- 1-5th. It is best given in powder, as follows:

℞ Pilocarpiæ muriat....................................gr j.

Sacch. lactis..........................................gr. xxv.

Div. in chart., No. V. M.

These powders may be given to the patient, with directions when to take them.

To prevent the occurrrnce of a chill, pilocarpin should be given hypodermically about fifteen minutes before the time when it would commence; if given by the mouth, an interval of half an hour is desirable, on account of the slower action of the drug when administered in this way. In cases where distinct prodromata, with which the patient is familiar, enable him to predict a chill, these will indicate when the medicine should be taken. In cases where there are no prodromata, it will be necessary to approximate, judging from the hours at which preceding chills have occurred. In those instances in which paroxysms come on at odd times, without any regularity, the patients may be advised to carry powders about with them, taking one whenever an attack seems impending. It is in this last class of cases that the administration by the mouth is especially convenient; the patient can have always with him a remedy which he can use at a moment's notice.

It is well to assist the action of the pilocarpin with warm coverings and a warm drink; it is noticeable that unpleasant salivation is least apt to occur in those cases in which diaphoresis is prompt and easy. Should the sweating cause the patient to feel cold and fatigued, a stimulant may be administered with propriety. In one case in which profuse diaphoresis continued longer than was desirable, it was checked promptly and without unpleasant symptoms with atropiæ sulphat., gr. 1-96th, administered hypodermically.

The advantages of this addition to the therapeutics of intermittent fever are sufficiently obvious. The physician, called to a patient in a chill, can at once give him relief. If the chill has just begun, the administration of pilocarpin will in most cases cause the paroxysm to abort, there will be no hot stage, and the patient will escape the exhaustion incident thereto; if the chill has been in progress for fifteen or twenty minutes before the pilocarpin is given, it will be cut short, and many of the patient's disagreeable sensations dispelled; but some fever will generally follow—this, however, will not range so high nor last so long as it would have done without treatment. In either case the tendency to the occurrence of another paroxysm will be much less than if the first had been allowed to run its course without interruption. Quinine may now be given, but need not be pushed to the induction of absolute cinchonism; for, if another chill should threaten to occur, it could with certainty be prevented with pilocarpin. In this way the patient escapes the unpleasant effects of large doses of quinine, the fever being none the less effectually cut short at once; paroxysms not being permitted to occur, exhaustion is avoided, and convalescence is easy and rapid. In cases where quinine, through idiosyncrasy, is contra-indicated, it may be left out of the treatment altogether, and entire reliance be placed upon pilocarpin. A large majority of cases of intermittent fever terminate without further treatment after the thorough abortion of a single paroxysm with pilocarpin; very few cases indeed, not five per cent., will continue long enough to require a third use of the remedy. One great advantage is that pilocarpin need not be used blindly; it is required only when a paroxysm is felt to be on the point of developing. If the necessity for the medicine does not present itself, the patient may be spared the inconvenience of taking it.

The power of the pilocarpin to cure intermittent fever entirely, by simply anticipating and preventing the occurrence of one or two paroxysms, is greater and more striking than will easily be believed by those unaccustomed to its use. I have histories of four cases which have already resisted quinine and other approved anti-malarial remedies for periods varying from two to four weeks, but which terminated after the decisive control of a single paroxysm with pilocarpin. The most recent of these recoveries took place three months ago, yet a relapse has not occurred in a single instance.

Vague and ill-defined malarial manifestations, headaches, neuralgia, etc., are very successfully treated by the administration of pilocarpin a few minutes before the time for their occurrence; the effect will be most satisfactory when the disturbance in question is attended with some rise of temperature, and is distinctly periodic.

No good results seem to follow the administration of pilocarpin during the hot stage; its efficacy appears to be limited to its power to prevent or break up that primary disturbance of the circulation which ushers in a paroxysm. It acts quite as well, however, in cases where the cold stage is not marked, if it is given early enough to produce diaphoresis before the fever is well declared.

After having witnessed its administration in nearly a hundred cases, the author feels justified in asserting that, in the doses required in intermittent fever, the action of pilocarpin is unattended with danger or discomfort; this assurance is certainly not superfluous, in view of the fact that many good authorities hold that its use in uræmic coma and convulsions (in which connection it is most familiar to the profession) has been followed by serious cardiac depression and pulmonary œdema.—GASPAR GRISWOLD, in *New York Med. Jour.*

